# Successful implementation of a wellness and tobacco cessation curriculum in psychosocial rehabilitation clubhouses

**DOI:** 10.1186/1471-2458-11-702

**Published:** 2011-09-14

**Authors:** Joseph GL Lee, Leah M Ranney, Adam O Goldstein, Anna McCullough, Sterling M Fulton-Smith, Nicole O Collins

**Affiliations:** 1 Tobacco Prevention and Evaluation Program, Department of Family Medicine, School of Medicine, University of North Carolina at Chapel Hill, CB 7595, 590 Manning Drive, Chapel Hill, North Carolina 27599 USA; 2 North Carolina Health and Wellness Trust Fund Commission, 7090 Mail Service Center, Raleigh, North Carolina 27699 USA; 3 Southern Regional Area Health Education Center, 1601 Owen Drive, Fayetteville, North Carolina 28304 USA

## Abstract

**Background:**

Tobacco remains a seemingly intractable problem for individuals living with severe and persistent mental illness. This study evaluated the implementation, technical assistance, and perceived impact of a model curriculum ("Learning About Healthy Living") to promote wellness and motivation to quit tobacco use in psychosocial rehabilitation clubhouses.

**Methods:**

We used semi-structured interviews (n = 9) with clubhouse staff (n = 12) and a survey of participating clubhouse members (n = 271) in nine clubhouses.

**Results:**

Fifty-eight percent of clubhouse participants completed surveys. Results showed tobacco users open to tobacco-free policies (62%) and perceiving more discussions about quitting tobacco with healthcare providers (69%). Analyses of staff interviews and member surveys revealed four key themes: (1) the curriculum was successfully implemented and appreciated; (2) technical assistance kept implementation on track; (3) adding wellness content and interactive components should enhance the curriculum; and, (4) the curriculum advanced other healthful policies and practices.

**Conclusions:**

Mental health settings are important locations for implementing programs to address tobacco use. In this real-world implementation of a model curriculum in psychosocial rehabilitation clubhouses, the curriculum tested well, was feasible and well-received, and suggests potential impact on tobacco use outcomes. Revision, dissemination, and a randomized controlled trial evaluation of the model curriculum should now occur.

## Background

Tobacco-related diseases are a primary cause of death for people living with severe and persistent mental illness (SPMI) [1] and lead to premature death an average of 25 years earlier than the general population [2]. There is a compelling need for interventions to address, in general, chronic disease prevention and, specifically, the normative nature of tobacco use among people with SPMI [3]. Evidence of the tobacco industry's targeting of people living with SPMI adds further urgency to implementing and evaluating interventions in mental health settings [4].

While smoking in the United States has declined over the past five decades, reductions are strikingly absent among individuals with serious and persistent mental illness (SPMI). While adults living with SPMI can and do quit when using evidence-based cessation treatment [5-7], albeit with higher relapse rates [8], smoking prevalence among individuals living with SPMI remains between 36% and 49% [9]. These large disparities in tobacco use persist because of slow progress in changing tobacco-related norms among mental health providers and clients as well as a historical reluctance by mental health providers to address tobacco addiction [10-12].

Focus groups with mental health providers and consumers have identified barriers to quitting among mental health consumers, including the normative nature of tobacco use in psychiatric campuses and clubhouses [11] and the use of tobacco as a coping strategy [13]. Since individuals with SPMI report reasons for wanting to quit tobacco use similar to the general population [14], a need exists to integrate cessation services into routine mental health care [6,12].

Clearly, more vigorous efforts are necessary to recognize and address tobacco use among individuals with SPMI [3,8]. Suggested approaches include: (1) combined counselling with pharmacotherapy treatment [8]; (2) policy change in mental health settings [11]; and, (3) integration of tobacco assessment and advice to quit with routine mental health care [15,16]. To address tobacco use in outpatient mental health settings, researchers at the University of Medicine and Dentistry of New Jersey (UMDNJ) proposed a model 26-week curriculum "Learning About Healthy Living: Tobacco and You" [17-19]. While Learning About Healthy Living is used in New Jersey's mental health system [20] and freely available online, evaluation of its dissemination or feasibility in other locations is limited to a single study by the curriculum's authors [19]. To date, no studies have documented its replicability or ability to be disseminated more widely. Similar approaches also using mental health clinicians for program implementation have shown promise in previous feasibility studies [19,21-24], as have programs focused on individualized assessment and goal setting [25-29].

The North Carolina Health & Wellness Trust Fund (HWTF), a state health promotion agency, and Southern Regional Area Health Education Center (SR-AHEC) developed a pilot program to disseminate the UMDNJ curriculum under the name "Breathe Easy, Live Well" (BELW) in nine voluntary, "outpatient" mental health settings in North Carolina (NC) that use the clubhouse model of psychosocial rehabilitation. Clubhouses provide a non-clinical, holistic approach to recovery for individuals living with SPMI. Clients (known as members) participate in their own recovery process through socializing and working in the clubhouse environment [30,31]. The International Center for Clubhouse Development http://www.iccd.org/ certifies clubhouses through a peer review process involving experienced staff and members. Previous research has shown that clubhouses have an existing interest in wellness programming [32]. The BELW curriculum was implemented with several important differences from the prior UMDNJ feasibility study [19]: (1) NC groups were open to all clubhouse members rather than smokers only; (2) SR-AHEC provided incentives to participating clubhouses; (3) staff did not routinely measure carbon monoxide levels; and, (4) group facilitators were lay staff and/or members instead of clinicians. These modifications were intended to make the program more feasible for mental health clubhouses across diverse settings in NC and many other states. The group was available to all members, regardless of tobacco use status, in order to be compatible with tenets of non-exclusivity among clubhouse programs. The attendance of non-tobacco users was also explicitly intended by the program designers to promote the group as a wellness group that covered tobacco, thus reducing barriers for tobacco users in pre-contemplative stages of change.

An independent evaluation team from the University of North Carolina at Chapel Hill worked with HWTF and SR-AHEC staff to define activities and program objectives through the use of a logic model [33]. The principles of utilization-focused evaluation [34] guided the evaluation team to provide actionable findings that could be used to improve the program in future iterations. Evaluators designed a process-oriented evaluation plan based on the program logic model to answer four key questions agreed upon by the implementation team:

(1) How was the curriculum implemented by clubhouse staff?

(2) How did clubhouse staff use and perceive technical assistance and training services?

(3) What were clubhouse members' barriers, facilitators, and levels of engagement in the curriculum?

(4) What were clubhouse staff and member perceptions about tobacco-related clubhouse norms and policy changes?

In addition to answering these questions, the process evaluation of the curriculum sought to determine the curriculum's feasibility for wider implementation across NC.

## Methods

### Program Description

The 26-week UMDNJ curriculum has two parts. The first promotes wellness and interest in quitting, and the second provides support to quit tobacco use. SR-AHEC personnel trained clubhouse staff and lead members at nine clubhouses. SR-AHEC personnel developed an in-person, two-day staff and lead member training to improve knowledge and skills in seven areas: history of tobacco use, tobacco products, nicotine addiction, pharmacology for tobacco dependence, health issues from tobacco and secondhand smoke, motivational interviewing, and the Learning About Healthy Living curriculum. The training concluded with a demonstration of group facilitation. Experts at UMDNJ served as consultants to assist SR-AHEC with development of the training and implementation of the 26-week curriculum.

SR-AHEC provided each clubhouse with a stipend of $7,500, paid in three parts, to incentivize clubhouse participation and cover program-related costs (e.g., healthy snacks, nicotine replacement therapy co-pays). Clubhouses and SR-AHEC provided incentives for members such as coffee, snacks, t-shirts, water bottles, wrist bands, and tote bags. These programmatic participation incentives are unrelated to the evaluation survey described below. The SR-AHEC team provided technical assistance through monthly in-person site visits, phone calls, email consultations, and conference calls. Common types of technical assistance are attached in a checklist (see additional file 1). During site visits, SR-AHEC staff would help lead the group, modelling strategies for group facilitation.

The SR-AHEC team expended $333,821 over two years on implementation, including the cost of a related projected promoting smoking cessation counselling among psychiatrists. The curriculum and groups started at four clubhouses in January 2009 and by November 2010 all nine clubhouses completed participation in the curriculum.

### Evaluation Approach

Consistent with evaluation best practice and the resources available for evaluation, the evaluation team used both qualitative and quantitative analysis, as appropriate, for different study aims [34,35]. The survey (see additional file 2) and interview protocol (see additional file 3) were developed directly from the program's logic model and resulting evaluation plan. A quantitative survey provided information on participants' attendance, reasons for participation, attitudes toward tobacco-related policies, perceptions of tobacco-related norms, and smoking status. The evaluation team deemed qualitative analysis better suited to identify how the program was implemented and to encourage open discussion with clubhouse staff about barriers, facilitators, and unforeseen outcomes.

### Survey of Participating Members

Evaluators designed a paper-based self-administered survey to capture information related to participants' smoking status ("Have you smoked a cigarette in the past seven days?"), level of program participation ("Approximately how many [Learning About Healthy Living] group meetings did you attend?"), perceptions of tobacco-related policies ("If the clubhouse did not allow tobacco use inside or outside of the clubhouse, would you still come to the clubhouse?"), views on tobacco norms ("Do you think members are more interested in quitting using tobacco because of the [Learning About Healthy Living] group?"), and perception of program impact on clubhouses ("Do you think members are more aware of the bad health effects of secondhand smoke because of the [Learning About Health Living] group?"). Stakeholders reviewed surveys and the evaluation team then tested for readability (grade level 5.5, reading ease 76.8% [Word 2007, Microsoft, Redmond, Washington]) [36]. A small wrist-band was provided to participants as an as a token of appreciation for filling out the survey. Clubhouse staff distributed the surveys to participating members and returned them to the evaluation team.

### Interviews with Clubhouse Staff

The evaluation team conducted in-person interviews with clubhouse staff who implemented the curriculum using a semi-structured (i.e., consistent questions with primarily open-ended response formats) interview protocol. Two authors (AM, JGLL) conducted interviews separately. The interviewer audio recorded with the participants' consent and obtained professional transcriptions for analysis. Interviews lasted between 30-60 minutes and were conducted in a private room at the clubhouse. All interviews were conducted after the completion of the curriculum.

### Analysis

#### Quantitative

Quantitative data analysis was conducted on the clubhouse member survey using SPSS 17 (IBM, Chicago, Illinois). Frequencies were reported from the survey responses. Respondents who reported zero participation (n = 8) as well as one unreadable survey were excluded. Since the clubhouses occasionally repeated or skipped weeks, self-reported attendance was calculated using 30 weeks.

#### Qualitative

Following the principles of utilization-focused evaluation [34], the evaluation team approached the data by allowing implementation-related themes to naturally emerge from interviews and applied deductive reasoning to answer key study questions about implementation, thus ensuring actionable information was provided to the program funder and implementation team. Guided by this and the study aims, the evaluation team coded staff interviews to identify specific answers to the study's four key questions through four deductive code groups consistent with the sections of the *a priori *evaluation plan established before the first wave of interviews. Deductive sub-codes from individual questions in the evaluation plan added depth to the coding system. For example, as part of the evaluation plan, the evaluation team sought to ascertain if the curriculum was presented as a "stop smoking" group or as a "wellness group" as intended. Under "implementation" a sub-code titled "frame" was thus used to capture descriptions of how the curriculum was presented to members. Such deductive coding delineated relevant data for key evaluation questions (e.g., was the curriculum implemented with fidelity?).

The evaluation team also believed that deductive coding alone provided inadequate depth for understanding the clubhouse context, program's value to staff, and complex barriers and facilitators of implementation. The evaluation team was particularly interested in a more nuanced approach to understanding the program given (a) the context of a mental health system where a paucity of resources may inflate perceptions of technical assistance provision and impacts of new programs and (b) the use of lay-staff and lead members instead of clinicians to lead the program, which could cause unforeseen changes in implementation not captured by deductive coding. By reading through all interviews and identifying themes within and across evaluation plan concept areas, additional inductive thematic codes were generated based on the content of the interviews using a grounded theory approach [37]. While this combination of deductive and inductive coding is not standard practice, *per se*, the evaluation team believed it to be warranted given the study's key evaluation questions. Interview data were coded in an iterative fashion with multiple waves of coding to capture emerging themes. One author (JGLL) coded interviews in MAXQDA 10 software (VERBI Software, Marburg, Germany). Each code was defined in a code memo. Multiple reviews of coded text segments yielded key themes.

The University of North Carolina Public Health-Nursing Institutional Review Board reviewed and approved the research plan as part of comprehensive program evaluation (09-1703). All interviews and surveys were conducted with informed consent.

## Results

### Survey and Interview Responses

Of the 271 participants in the nine clubhouse programs, 58% returned valid surveys at the end of the curriculum. More female than male participants responded (Table 1), with a mean age of 47. Forty-four percent of survey respondents reported smoking in the last seven days, and 10% reported using smokeless tobacco in the last seven days. The evaluation team completed interviews with staff (n = 12) at all nine clubhouses, and coding resulted in 748 coded text segments across 78 codes (22 deductive, 56 inductive). The findings from the survey and interviews are presented by the four evaluation question domains.

**Table 1:** Survey demographics and description of program, by tobacco use status

	Non-tobacco users (n = 84)	Tobacco users (n = 73)
**Gender**		

Female	62%	48%

Male	38%	52%

Overall	53%	47%

**Age**		

Mean	47	46

Range	22-76	20-75

**Description of Program**		

Open and helpful group activity	69%	69%

No pressure to join	43%	59%

To help me be healthier	76%	70%

To help me quit	-	82%

Note: percentages do not total 100 as multiple responses could be selected.

#### 1. Implementation: the program was appreciated and successfully implemented with general fidelity

The curriculum was implemented in all nine clubhouses. Eight of the nine clubhouses successfully implemented the program. Of the clubhouses that more successfully implemented the program, several factors were salient in interviews: (a) supportive clubhouse environments; (b) pre-existing interest in adopting a wellness curriculum or program; and, (c) staff time and support. In one clubhouse, the evaluation team determined implementation to be less successful given challenges reported by staff, and no smokers were reported to be participating by the end of the curriculum. This divergence from the other clubhouses was determined to be a result of internal factors at that specific clubhouse (e.g., [a] limited institutional support, [b] re-organization, and [c] a staff member who was assigned to facilitate rather than who volunteered or sought out the opportunity). Staff member views at this one clubhouse also contradicted the program goal, for example, asserting, "the one freedom they have is to go out the back door and go smoke."

##### Program recruitment

Fidelity to the intended framing of the group as a wellness (versus tobacco cessation) program was mixed. Interviews indicate that the curriculum was presented to members as a wellness program in about half of clubhouses and as a "quit tobacco" program in the other half of clubhouses. Member surveys indicate that members viewed the program being described as both to promote health and to help quit using tobacco (Table 1).

##### Member participation

**Figure 1:**
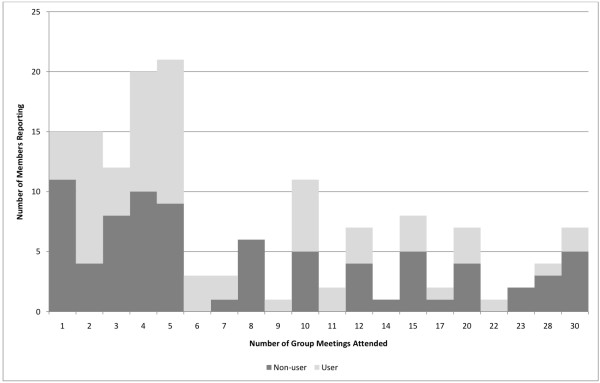
**Self-reported participation in 26-30 week BELW curriculum**.

Members participated in a median number of five group meetings (range 1 to 30) (Figure 1). Seventy-five percent of participating members reported participating in 12 or fewer of the 26-30 sessions, and participating members selected a number of reasons for their participation and perceptions of reasons for other members' nonparticipation (Table 2).

**Table 2:** Members' reasons and perceptions relating to participation by tobacco use status

	% of non-tobacco users reporting (n = 84)	% of tobacco users reporting (n = 73)
**Self-reported reasons for participating**		

I wanted to learn about being healthy	82%	67%

It seemed interesting	61%	51%

I wanted to quit using tobacco	-	66%

I had nothing better to do	16%	15%

**Participants' perceptions of reasons for non-participation**		

They were not interested	69%	81%

Group met at the wrong time	18%	10%

Other reasons	17%	14%

Note: percentages do not total 100 as multiple responses could be selected.

##### Staff and member views of program

During the interviews, staff intimated that they had been concerned about the viability of the program at its start. As the interview progressed, however, staff indicated that their scepticism had turned to surprise, describing how the curriculum had provided a sense of camaraderie among members and generated energy for other healthful changes in the clubhouse. The curriculum seemed to provide a needed space for discussion, the sharing of challenges, and working on strategies used for overcoming them. New friendships, walking buddies, and an interest in a broad spectrum of chronic disease prevention activities emerged from this program, and staff saw it as an added benefit and service of the clubhouse. Interviewees generally noted positive outcomes from the program, some of which were unanticipated.

"*I didn't think we were going to see any [effects], to be honest. I think that they came in reluctant, and they were like, 'You're taking something away from me..' [...] 'I'll come in your class, but I'm keep[ing] smoking.'" *(Interview 6)

"*So I've noticed that people really appreciate the space to check in with each other. That's been the biggest benefit*." (Interview 4)

"*Just how well the...how many that did quit and then the reductions. And then what came out of it with the walking group. That was unexpected. It was a really happy unexpected thing." *(Interview 9)

Such energy around wellness issues seemed to achieve the programmatic goal of increasing awareness of the harms of tobacco and increasing motivation to think about and attempt quitting. Energy stemmed from having the opportunity to engage around wellness issues, and staff perceived the program as a whole to have a positive impact on the clubhouse and members.

"*It seems like everybody, everybody did something to improve their life. They did something, whether it was eat different or walk a mile every day. They all found something to improve their way of life*." (Interview 8)

"*I think it's had a positive impact. People look forward to coming here, now that there's a health and wellness component to the clubhouse*." (Interview 6)

"*[Members] look forward just to that time that we're all sitting in a group and talking about something together*." (Interview 3)

#### 2. Training and technical assistance: vital to program implementation

Perhaps related to initial scepticism about the program, staff were very pleased with having adequate assistance and resources that were provided in a non-judgmental way. Such positive experiences with support in the context of a state mental health system rocked by re-organizations, shifting priorities, and shrinking budgets [38] may have influenced these views. The evaluation team included an open-ended question on assistance that helped make the program successful ("What were the three most important resources for making Breathe Easy, Live Well happen at [clubhouse name]?"). One of the most salient themes to emerge out of responses were discussions of the quality and utility of the technical assistance. The quality of technical assistance was matched by the importance of having someone keep track of the project. Without this type of assistance, the busy clubhouse environment may not have implemented the program as successfully. Staff believed that the program's success was primarily the result of the SR-AHEC technical assistance team, which received universal plaudits from clubhouse staff and helped keep implementation on track. Other resources such as the trainings and the toolkit were also instrumental.

"*One, for me, one is training. [SR-AHEC staff member] and his assistant were A-plus excellent, always attending to our needs, always sending us emails, letting us know when they were coming...being accommodating as we need them to be, but not in our face, and I think that helps*." (Interview 6)

"*[I]t was kind of like an 'overseer' to come in and touch base which was good for all of us. It kind of made [us] think, 'Okay, well [SR-AHEC staff person] might be here in two weeks. We better get on our toes, and we better start.'*" (Interview 1)

"*It wouldn't have went over [without the SR-AHEC support]. It would have been one of those things that was a nice idea and then fell to the wayside*." (Interview 9)

"*If they had just gave it to me and been like you do this every week, no, 'cause we got five other things going at one time." *(Interview 7)

#### 3. Curricular barriers and facilitators: more interactive and wellness focused content needed

Despite generally positive views of the program and quality technical assistance, interviewees indicated some challenges with the curriculum, requiring them to make changes to activities, add material, and integrate other wellness resources. The evaluation team noted two concurrent themes relating to the curriculum: interviewees noted adherence to the curriculum's intended strategy, but many tactics for implementation were adapted to better fit group members' needs. Most modifications fell into two categories: (a) adding wellness content and (b) making the curriculum's activities more interactive. While tobacco-specific information was appreciated and reportedly interesting, staff felt compelled to provide resources about diet and exercise for non-smoking participants.

"*For the ones that kept coming, we pretty much had to always include a wellness, a healthy eatin' [component] as part of the conversation. How to read the back of packages and understand sugar content and stuff like that. You had to put something about wellness in there*." (Interview 7)

"*I mean there was some wellness stuff in the curriculum book but not a lot and I think just because of the makeup of our group, it wasn't the most applicable*." (Interview 4)

"*[With] the planning and all that, it makes it kind of a little bit challenging for us to incorporate it to the people, because majority of our group actually are the non-smokers... then that kind of bored other people when we talk about nicotine and how to less the nicotine. So, then we started [saying] 'okay these are the techniques' and then we just went to mostly giving towards the needs of our participants, who said they want to lose weight, so they be more active: 'let's talk about how do we lose the weight that we all really plan to lose.'*" (Interview 5)

Staff also modified the curriculum to make it more interactive and engaging for members by trying to make material come alive and avoid what seemed like rote, individualized worksheets. Such staff efforts seem consistent with principles of health education and in line with the motivation and peer-support theories underpinning the curriculum's design.

"*Some of those chapters didn't really provide, if you go right through the toolkit, didn't provide people to speak of their own experiences. It was kind of an A, B, or C, check one, and if that doesn't apply then maybe people wouldn't want to share so much*." (Interview 8)

"*[In the curriculum,] I feel like there's generally two or three or four exercises, written exercises ... and I feel like the best approach would be creating some model where it's creating a group discussion as opposed to writing out our answers*." (Interview 4)

"*If there's more maybe kind of interactive kind of things that we could do next time when they redo the thing; more interactive things to give them to use*." (Interview 9)

Substantial barriers existed for the promotion of pharmacotherapy for tobacco use cessation. Specifically, staff reported barriers to accessing health professionals for pharmacotherapy support, discomfort with the promotion of pharmacotherapy, and challenges dealing with side effects. Interviewees perceived little success in promoting the use of pharmacotherapy for tobacco cessation. Moreover, promotion of medication runs somewhat counter to the member-centred, non-clinical work of clubhouses. Additional questions revealed that staff felt overwhelmed by the complexity of drug interaction, dosing, and side effects. Little support was available from medical providers as there were few links between medical providers and clubhouse staff around cessation. Staff also reported barriers due to complicated and limited insurance coverage of nicotine replacement therapy and smoking cessation medications. However, participating members reported that they believe more discussions were being held between clubhouse members and their medical providers because of the group (Table 3).

**Table 3:** Tobacco users' perceptions of impact

	% tobacco users reporting
**Perception of members talking to medical providers more about quitting because of the group (n = 71)**	

Yes	69%

No	11%

Do not know	20%

**Perception of more awareness of bad health effects of secondhand smoke among members (n = 72)**	

Yes	82%

No	8%

Do not know	10%

"*We had a conference call with the pharmaceutical thing, but that was really kind of over our head*." (Interview 9)

"*It's the clubhouse motto, it's not something we do, so I don't push that too [much] further*." (Interview 6)

"*When we were quitting and reducing cigarettes and tobacco usage, the symptoms of their illness or ... medications, will increase ... And that was very scary for me*." (Interview 8)

#### 4. Norms and policies: staff and members leveraged the groups and curriculum to advance healthful policies

Clubhouse members who used tobacco in the last seven days overwhelmingly reported (71%) that they quit or cut down tobacco use because of the curriculum. Moreover, interviews suggesting that the curriculum had helped change the discourse around tobacco and provided additional social support for quitting. Such normative changes could create sigma for tobacco users; however, staff (some of whom themselves smoked) indicated that the curriculum emphasized de-normalizing and overcoming tobacco use by focusing on motivation and support rather than by stigmatizing use. Interviewees and survey respondents reported increased talk about and awareness of the harms of tobacco use that went well beyond the participating members (Table 3).

"*Yeah, they are telling each other you know, that's not very good, or kind of talking each other down, still using buddy systems. When people come into the clubhouse it's one of the first programs that they really tell somebody else about. [...] They're finding different ways to cope with their illnesses rather than just sit there and smoke*." (Interview 8)

"*Since the program, I think they...it's like they realize how it's not good for you and they...I don't want to say they're shunned because they're smoking, but it's not...like you don't hear them fussing that they have to go outside to smoke, that kind of thing." *(Interview 9)

"*And I think when they see other members who participate in the program, or they see me, it kind of has to have that effect, like, 'I'm trying to quit!' [laughter] 'I'm going to come to your class, you know. I'm not ready to come...' but it's like they have that, there's something and they're looking at you like 'You're a part of that smoking wellness program.' And the people who have quit, they are a big part of it too, because they're looking at them and saying, 'If they can do it, I can do it.'*" (Interview 6)

Additionally, changes in norms manifested themselves in physical spaces and practices or policies. Clubhouses adopted healthful policies and practices (e.g., walking groups, sponsored YMCA [i.e., gym] memberships, tobacco-free areas) that staff attributed to the energy generated by the program.

*"[A]t one time there was people that would smoke out on the front porch. Well, the non-smokers, just kind of like, took over the porch. [Chuckle]*" (Interview 9)

"*We sort of used the fact that we were bumping up our wellness programming and we were doing Breathe Easy, Live Well to help us move the smoking into some set locations rather than all around. I think having it tied to a wellness program rather than just a seemingly random decision helped make it palatable*." (Interview 4)

"*With that front porch, as soon as you come you see cigarette butts. It made it hard for us before to just [say], 'hey, you, go to the smoking area,' 'smoke there,' 'do not drop your cigarettes in here.' One reason [for our smokefree front door policy] is that we've been talking about smoking [in the groups] for almost a year*." (Interview 5)

Clubhouses reported that the energy generated by the program also contributed to food service and physical activity changes, which were often easier to talk about and engage on than tobacco issues.

"*I think nobody stopped smoking in our group but they really thought about it. It made them think and really talking about healthy eating[, which] they loved*." (Interview 3)

"*You can just look around and you can see bottles of water everywhere where a year ago you just saw a soda bottle*." (Interview 1)

"*We had a walking group that came out of the meeting and walk every day at 1:15, almost every day*." (Interview 2)

Three clubhouses adopted smoke-free porch policies. One clubhouse restricted smoking to certain times and prohibited staff from smoking with members. Additionally, over half of members surveyed reported that they believe clubhouse members are interested in new no-tobacco areas (Table 4). Impressively, most tobacco-using participants (84%) indicated they would continue to attend the clubhouse if tobacco use were not allowed inside or outside and that a tobacco-free policy would make quitting easier.

**Table 4:** Tobacco users' perceptions of tobacco-free policies

	% tobacco users reporting
**Would a tobacco-free policy indoors and outdoors make quitting easier? (n = 68)**	

Yes	70%

No	18%

Do not know	12%

**Are members of the clubhouse interested in new tobacco-free areas? (n = 73)**	

Yes	62%

No	19%

Do not know	19%

**If the clubhouse did not allow tobacco use inside or outside of the clubhouse, would you still come to the clubhouse? (n = 70)**	

Yes	84%

No	7%

Do not know	9%

Interviewees reported that while policy change would be difficult in the short-term given the history of mental health services, there is long-term hope. In particular, interviewees noted that the process of changing norms through programs like BELW is needed to transition to tobacco-free policies.

Staff views about policy change were mixed. An abrupt implementation in a tobacco-free policy would lead to "*Tyranny! It'd be chaos!*" as one staff interviewee noted with a smile. Other staff members noted that while tobacco-free policies would be difficult to implement, members would likely adapt. Interviewees viewed the lack of policy adoption as a result of the many pressing concerns clubhouse staff and management face.

"*It's just been around so long we just haven't got there yet. There's too many other things you worry about*." (Interview 8)

While staff viewed 100% tobacco-free policies to be a lofty goal, they saw group wellness programs like BELW as a way to support quitting and build long-term change. One staff person, who above noted the tyranny and chaos of a tobacco-free policy, upon further probing suggested a path forward:

"*Just what we're doing, I think, having a group, word of mouth. And I think more members are going out, and they're saying, 'Hey, I'm in the smoking class, and it's helping me.'*" (Interview 6)

## Discussion

A structured group approach to promoting healthful living and tobacco cessation can be an important tool in addressing the high tobacco use prevalence among people living with SPMI. Like Williams, *et al *[19], we found that the Learning About Healthy Living curriculum was well appreciated and feasible to implement as part of BELW. Moreover, we implemented the curriculum using non-clinician clubhouse staff in a real-world state health promotion effort. The program was successfully used to generate momentum for advancing tobacco and wellness policy changes in clubhouse settings.

Our evaluation, however, found several areas of concern and needed improvement for the curriculum. Staff consistently modified the curriculum to make it more interactive and focus attention on discussion rather than the written worksheets. Since the groups were open to all members instead of just smokers, content areas outside of tobacco use and cessation seemed inadequate, and staff frequently supplemented with their own nutrition and physical activity material. Promotion of pharmacotherapy fell flat, likely due to the use of non-clinical facilitators.

Future efforts that expand upon the curriculum and increase participation rates should include improving interactivity, providing consistent wellness materials, and integrating both into the group routine. A critical component of curriculum implementation may be to ensure that high levels of monitoring, technical assistance, and support are available in future programs. Staff reported that technical assistance provided useful skills as well as important motivation to keep implementation on track in a busy mental health setting. Scaling up the program for wider implementation may require finding a lower-intensity level of technical assistance and reducing participation incentives for clubhouses due to available funding resources. The effect of those reductions may change program feasibility and adoption. Further investigation of necessary technical assistance levels is needed.

This evaluation has a number of limitations. Given their self-selection to take part in this pilot program, participating clubhouses may not be representative of other clubhouses. The members who responded to our survey may be different from those who stopped participating in the group and therefore did not respond to the post-group survey. The inclusion of non-tobacco users in the curriculum limits comparisons to previous implementation solely for tobacco users. Insufficient resources existed to conduct an impact evaluation, so the evidence for a quantifiable impact on health behaviour is limited. One clubhouse simultaneously participated in a separate wellness assessment project that may have influenced interview responses [23,24].

Nonetheless, our process evaluation provides important feedback from real-world implementation that can improve the existing curriculum for future use and will be important to efforts to refine or develop wellness promotion strategies in clubhouse settings. The inclusion of non-tobacco users may facilitate participation by tobacco users in pre-contemplation stages of change. This evaluation also informed next steps in program development and implementation: evaluation results helped SR-AHEC develop a 15-week version of the curriculum with more wellness content. Implementation and testing are underway in 14 non-clubhouse psychosocial rehabilitation centres.

Our finding that providing a time and place for discussions of wellness contributed to policy changes merits special attention in the mental health system, which has been challenged by limited adoption of smoke- and tobacco-free policies. Wellness group efforts like the one we evaluated can potentially be an important part of broader strategies to diffuse health-related policy adoption across the mental health system. Such efforts are increasingly important given the burgeoning recognition of the power of policies to advance behaviour change [39], recognition which is borne of social-ecological approaches to improving health by changing individuals' environments [40]. The value of programs like BELW and the Learning About Healthy Living curriculum may reside at multiple levels of the social ecological framework: improving individual knowledge about healthy behaviours, providing support within an immediate peer group, and contributing to healthful policy changes in the daily environment of individuals living with SPMI. Future consideration of these multiple levels of impact is warranted as researchers move forward in quantifying the impact of wellness programs in mental health settings.

## Conclusions

Our findings suggest that the Learning About Healthy Living modified program is appreciated and well-received. With modifications, this and other programs can help address endemic tobacco use in mental health settings and among people living with SPMI. The original UMDNJ curriculum complements other state-wide efforts by focusing on education and normative change in an underserved population [19]. Mental health consumers have disproportionately high rates of tobacco use and can benefit from wellness programs that address other health issues in combination with tobacco cessation treatment, particularly if such programs can be leveraged to support lasting policy changes. This curriculum should receive further impact evaluation through a randomized controlled trial.

## Competing interests

Dr. Goldstein has received unrestricted educational funding from Pfizer Pharmaceuticals to support dissemination of comprehensive tobacco cessation programs.

## Authors' contributions

All authors participated in the design of the study. JGLL conducted interviews, coded interviews, and conducted all data analyses. AM conducted interviews. All authors provided critical feedback and revisions to the text and approved the final text.

JGLL holds an MPH, LMR holds a PhD, AOG holds an MD and MPH, AM holds an MSW and MSPH, SMFS holds an MHA, and NOC holds an MA.

## Pre-publication history

The pre-publication history for this paper can be accessed here:

http://www.biomedcentral.com/1471-2458/11/702/prepub

## Supplementary Material

Additional file 1 **Technical assistance checklist**. PDF of list of commonly provided technical assistance subjects. Click here for file

Additional file 2 **Survey**. PDF of survey used for participating member (i.e., client) survey. Click here for file

Additional file 3 **Interview protocol**. PDF of interview protocol used for staff interviews. Click here for file
