# Supplementary material for: Successful implementation of a wellness and tobacco cessation curriculum in psychosocial rehabilitation clubhouses
Source: BMC Public Health. 2011 Sep 14;11:702. doi: 10.1186/1471-2458-11-702 (PMC3184072; doi:10.1186/1471-2458-11-702)
Supplement: Additional file 1 — Technical assistance checklist. PDF of list of commonly provided technical assistance subjects. [file 1471-2458-11-702-S1.PDF]

Technical Assistance Checklist: Breathe Easy, Live Well

Clubhouse: _______________ **Date**: ____________________

I discussed:

Recruiting and Retaining Members

- ☐ How to interest members to join the group
- ☐ How to interest tobacco users to join the group
- ☐ How to integrate new members into the existing group
- ☐ How to ensure non-tobacco users feel included in the group
- ☐ The importance of framing the group as a *wellness group*, not a quit tobacco group

Promoting Interaction

- ☐ Staff person's comfort with facilitating the group meetings
- ☐ Staff person's comfort using motivational interviewing
  - ☐ I modeled techniques for staff.
  - ☐ The staff person demonstrated techniques back to me.
- ☐ How to promote interaction between members in the group
  - ☐ I modeled techniques to improve interaction.
  - ☐ The staff person demonstrated techniques back to me.

Promoting Healthy Lives

- ☐ How to balance promoting positive change for everyone (tobacco users and non-users)
  - ☐ Content questions about tobacco cessation
  - ☐ Content questions about physical activity
  - ☐ Content questions about nutrition (including healthy beverage choices)
  - ☐ Content questions about policy changes
  - ☐ Content questions about handling stress
  - ☐ Content questions about regular doctor visits
  - ☐ Content questions about appropriate support networks

Policy Adoption

- ☐ Possible policies relating to tobacco that the clubhouse could adopt
  - ☐ Smoke-free areas
  - ☐ No staff smoking with members
- ☐ Possible policies relating to physical activity/nutrition that the clubhouse could adopt
  - ☐ Healthy walking clubs
  - ☐ Food, snack, and water options
  - ☐ Edible gardens

Communication with Healthcare Providers

- ☐ How to facilitate communication with healthcare provider
  - ☐ How to refer questions on pharmacotherapy and side effects to healthcare provider
  - ☐ I modeled the techniques for staff
  - ☐ The staff person demonstrated techniques back to me

- ☐ **Other(s):** ____
- ☐ **Follow-up Required** (*note on back*)

Definitions and Prompts for TA Provision: Breathe Easy, Live Well

Recruiting and Retaining Members

When gaining interest in the group…

- Start discussing Breathe Easy, Live Well program 1-2 months before actual program begins
- Emphasize that the group supports wellness, with a focus on tobacco use
- Use incentives to gain interest
- Make the first meeting an event
- Personally address clubhouse members about Breathe Easy, Live Well

When interesting tobacco users to join the group…

- Do not pressure them to join
- Stress that this is not a cessation group—you don't have to commit to quitting
- Mention that the primary focus is all about supporting other members, and that other members would benefit with extra support

When integrating new members…

- Have staff suggest that new members to the clubhouse try out the program
- Offer Breathe Easy, Live Well as part of clubhouse activities
- Encourage new members to come whenever they would like

When making sure that non-tobacco users feel included…

- Use examples of other behaviors as much as possible
    - Ex. The dangers of overeating to go along with the dangers of cigarettes

When discussing the importance of framing the group as a *wellness* group…

- Constantly refocus on overall wellness—this is not just a tobacco program
- Constantly emphasize the wellness part of the program and why it needs to be inclusive

Promoting Interaction

When discussing staff's comfort with facilitating meetings…

- Model examples of how to integrate questions and discussion points in each section

When discussing staff's comfort with motivational interviewing…

- Make sure staff knows to ask open questions, and not give advice (unless asked)
- Encourage staff to integrate own style into the group, and if possible, use ideas outside of the toolkit

When discussing promoting interaction between members in the group…

- Encourage asking questions throughout the content

Promoting Healthy Lives

When discussing how to balance promoting positive change for all members…

- Encourage focusing on one little change at a time
    - Use a baby step approach to avoid failures, Start slow

Policy Adoption

When noting possible tobacco-related policies that could be adopted…

- Discuss with directors about any possible issues during recruiting
- Assess current policies and consequences for violators
    - Give examples of challenges for those trying to quit based on current policies

When suggesting possible policies or activities that the clubhouse could adopt…

- Suggest discussion topics for group meetings such as:
    - What types of positive changes are you looking for?
    - What would make living healthier easier for you?

Communication with Healthcare Providers

When discussing how to facilitate communication with healthcare providers…

- Emphasize contact PCP if interested in quitting

Other suggestions

- Make sure there is constant staff encouragement towards members
- Make sure that staff encourages members discuss increased activity opportunities, healthier food choices, etc.
    - Really be influential in the group
    - Find out if there could be anything related to policy change
