# Supplementary material for: Successful implementation of a wellness and tobacco cessation curriculum in psychosocial rehabilitation clubhouses
Source: BMC Public Health. 2011 Sep 14;11:702. doi: 10.1186/1471-2458-11-702 (PMC3184072; doi:10.1186/1471-2458-11-702)
Supplement: Additional file 2 — Survey. PDF of survey used for participating member (i.e., client) survey. [file 1471-2458-11-702-S2.PDF]

We are inviting you to participate in a short survey of the "Breathe Easy, Live Well" group (the group about quitting tobacco) in your clubhouse. The purpose is to help improve this program for adults like you across North Carolina. If you join this survey, we will ask you some questions about "Breathe Easy, Live Well."

Joining the survey is up to you. If you do not join, you will not lose any benefits you have at the clubhouse. We will not collect any personal information about you.

It should take you about less than ten minutes to finish the questions for this survey. If you do not want to answer some of the questions, you can leave them blank. You can stop answering questions at any time.

This survey will help us learn new information and may help people in the future. You may or may not receive any direct benefit from being in the survey. This survey has no or little risk. You can stop this survey at any time.

About this study:

Title of Study: Breathe Easy, Live Well Pilot Project Process Evaluation

Principal Investigator: Adam O. Goldstein, MD, MPH
UNC-Chapel Hill Department: Family Medicine
UNC-Chapel Hill Phone number: 919-966-4090
Funding Source: North Carolina Health and Wellness Trust Fund

Study Contact telephone number: 919-966-8948
Study Contact email: tpep@med.unc.edu

Finishing the survey means you give permission for us to use your answers with others who complete the survey. We will combine answers in a report.

1. What is your gender?
   [Circle one choice.]

   a. Female
   b. Male

2. What is your age?

   _______ years

3. Have you smoked a cigarette in the past seven days?

   Yes ☐ No ☐

4. Have you used smokeless tobacco in the past seven days?

   Yes ☐ No ☐

5. How did you first hear about the "Breathe Easy, Live Well" group (the group about quitting tobacco)?
   [Circle one choice.]

   a. Clubhouse Staff
   b. Clubhouse Member
   c. Flyer or Sign
   d. Other: [*If so, who or what?*] ________________________

6. When you <u>first</u> heard about “Breathe Easy, Live Well,” how was it described?

	**Yes**	**No**
Open and helpful group activity…………………………	☐	☐
No pressure to join……………………………………..	☐	☐
To help me become healthier……………………………	☐	☐
To help me quit using tobacco…………………………..	☐	☐

7. Why did you choose to participate in the “Breathe Easy, Live Well” group?

	**Yes**	**No**
I had nothing better to do………………………………..	☐	☐
It seemed interesting…………………………………..	☐	☐
I wanted to learn about being healthy…………………..	☐	☐
I wanted to quit using tobacco…………………………..	☐	☐

8. Approximately how many “Breathe Easy, Live Well” group meetings did you attend?

_____ group meetings

9. Why do you think other clubhouse members did not take part in the “Breathe Easy, Live Well” group?

	**Yes**	**No**
They were not interested	☐	☐
Group met at the wrong time	☐	☐

Other reason [*write the reason*] ____________________________________
____

10. Do you think members in your clubhouse are more interested in creating new no-tobacco areas because of the “Breathe Easy, Live Well” group? (For example, a no-smoking area by the front door)

Yes	No	Do not know
☐	☐	☐

11. Do you think members are more interested in quitting using tobacco because of the “Breathe Easy, Live Well” group?

Yes	No	Do not know
☐	☐	☐

12. Did going to “Breathe Easy, Live Well” group meetings cause you to cut down or quit using tobacco?

Yes	No	Do not know
☐	☐	☐

13. Do you think members who use tobacco talk to their doctors more about quitting tobacco use because of the “Breathe Easy, Live Well” group?

Yes	No	Do not know
☐	☐	☐

14. Do you think members are more aware of the bad health effects of secondhand smoke because of the “Breathe Easy, Live Well” group?

Yes	No	Do not know
☐	☐	☐

15. If the clubhouse did not allow tobacco use inside or outside of the clubhouse, would it help you to quit or reduce your tobacco use?

Yes	No	Do not know	I do not use tobacco
☐	☐	☐	☐

16. If the clubhouse did not allow tobacco use inside or outside of the clubhouse, would you still come to the clubhouse?

Yes	No	Do not know
☐	☐	☐

The end

Thank you for taking part in this survey.
