# Supplementary material for: Successful implementation of a wellness and tobacco cessation curriculum in psychosocial rehabilitation clubhouses
Source: BMC Public Health. 2011 Sep 14;11:702. doi: 10.1186/1471-2458-11-702 (PMC3184072; doi:10.1186/1471-2458-11-702)
Supplement: Additional file 3 — Interview protocol. PDF of interview protocol used for staff interviews. [file 1471-2458-11-702-S3.PDF]

Interview Guide for Clubhouse Staff

Breathe Easy, Live Well Pilot Project Evaluation, Wave I

Introduction

Hello, I'm __________ from the Tobacco Prevention and Evaluation Program at the University of North Carolina at Chapel Hill. We are working with Todd and AHEC to evaluate Breathe Easy, Live Well and the "Learning About Healthy Living" curriculum. We are interested in your experience with Breathe Easy, Live Well. In particular, we are interested in what parts of Breathe Easy, Live Well worked well for you and what we can do to improve the program.

Your participation in this interview is voluntary and you can stop at anytime. Also, the interview is confidential. This means we will not share your name or affiliation. The interview will take approximately an hour to complete, and you will receive a small gift, a water bottle as a thank you for your time. Would you be willing to participate in the interview?

[If the response is no, thank the person for their time and offer contact information if they change their mind.]

[If the response is yes – continue below:]

I find it helpful to record interviews so I don't miss important information in my notes. May I record our conversation?

[If yes, initiate audio recording device.]

[Restate:] Do you understand that your participation in this interview is voluntary and you can stop at any time?

[If non-verbal response, ask for verbal response.]

Again, we will not share your name or identity in our reports. May I record our conversation?

Questions

[Frame] First, I would like to talk about your experience with Breathe Easy, Live Well.

1. **What was your role in running Breathe Easy, Live Well?**
2. **What strategies did you use to gain member participation before the Breathe Easy, Live Well group met for the first time?**
3. **Which of these strategies worked best with your members?**
4. **How did you describe Breathe Easy, Live Well to members in your clubhouse?**

   [Prompt: Pretend I'm a member; tell me about it]

5. **Tell me about how you ran the group. For example, what was your process for getting the group started?**

   [Check if mentioned; probe if not mentioned:

   ☐ Introduction and Ground Rules (i.e., open and affirming w/clear ground rules set)

   Tell me about the introduction to the group and any ground rules.

   ☐ Healthy living

   Tell me about starting and running the parts on healthy living.

   ☐ Getting ready to quit

   Tell me about starting and running the parts on quitting tobacco use.

   ☐ Mutual Support (members encourage one-another)

   Tell me about getting members to support and encourage each other.

6. **Why do you think members continued to participate in Breathe Easy, Live Well?**
7. **What do you think are the biggest reasons why other clubhouse members did not participate?**
8. **Which parts of Breathe Easy, Live Well did members seem more interested in?**

   [Check if mentioned; probe if not mentioned:

   ☐ Health effects

   How interested were members in learning about the health effects of tobacco?

☐ Managing Stress

How interested were members in learning about managing stress?

☐ Food choices

How interested were members in learning about making healthy food choices?

☐ Quitting tobacco use

How interested were members in learning about quitting tobacco use?

☐ Pharmacotherapy for tobacco use cessation

How interested were members in learning about nicotine replacement therapy or medicines to stop using tobacco?

[FRAME:] Now, I want to talk a little about how you ran the Breathe Easy, Live Well group.

9. **Think about the things you learned in Todd's trainings. Which were most helpful in running the group?**
10. **What other techniques or skills that you already had were helpful in running the group?**
11. **Tell me about how you used motivational interviewing in the group?**

    [Probe if not used or unsure:

    What are some reasons you didn't use motivational interviewing?

    What would you have needed to use motivational interviewing?

[FRAME:] Now, I would like to ask you about the effects of Breathe Easy, Live Well.

12. **What impact do you think Breathe Easy, Live Well had on clubhouse members?**

    [Prompt:

    ☐ What changes have you noticed in the way members and staff talk about tobacco in the clubhouse?

    ☐ How has Breathe Easy, Live Well changed staff and member interest in clubhouse tobacco policies?

☐ For members who participated in Breathe Easy, Live Well, how do you think the experience affected their thinking about quitting?

☐ How did Breathe Easy, Live Well affect staff and member confidence in being able to quit?

13. **How do you think Breathe Easy, Live Well will be used in the future at [clubhouse name]?**

    [Probe: If not used, what are the reasons that the curriculum will be continued or discontinued?

[FRAME:] I would like to talk about the help you received running Breathe Easy, Live Well.

14. **What were the three most important resources for making Breathe Easy, Live Well happen at [clubhouse name]?**

    [Note items **_________________, ___________________, ___________________**

    [PROMPT:] Tell me more about each of those.

    [Check if mentioned; probe if not mentioned:

    ☐ Tell me more about how you used the Breathe Easy, Live Well Clubhouse Training?

    ☐ Tell me more about the help from Todd via phone and e-mail?

    ☐ Tell me more about the help from Todd in person?

    ☐ How about the weekly sign-in sheet and initial and quarterly assessment forms?

15. **You said these three things were most important ____, ____, ____. What would it be like to run Breathe Easy, Live Well without each of those?**

    [Prompt if not mentioned previously:

    ☐ How did you use the $7,500 stipend?

    ☐ What if Todd only provided help via phone and e-mail instead of visiting?

16. **What were some things that made running Breathe Easy, Live Well harder?**

17. **Imagine Breathe Easy, Live Well will be used on a larger scale, in other clubhouses or other mental health treatment settings, what additional training and help might be useful to people running this group in another setting?**

[FRAME:] I now have one question about working with medical providers.

18. **Tell me about how medical providers are currently involved in promoting nicotine replacement therapy and medication to help members stop using tobacco?**

    [Probe if problem:] What needs to happen to overcome some of these challenges?

[FRAME:] We are nearing the end, but first I want to ask about tobacco policies at [clubhouse name].

19. **How do you think members might react if the clubhouse ever went 100% tobacco-free (meaning no tobacco use inside or outside on the clubhouse grounds)?**

20. **Can you see a time when [clubhouse name] might adopt a 100% tobacco-free policy (again meaning no tobacco inside or outside)?**

    [If no, continue to next question.]

    [If yes, probe:

    - ☐ What are some reasons why there is not currently a 100% tobacco-free policy?
    - ☐ What skills or resources would the clubhouse need to implement a 100% tobacco-free policy?

21. [SKIP IF YES to previous question] **What do you think keeps the clubhouse from adopting a 100% tobacco-free policy (again, I mean this to include both inside and outside)?**

22. **Tell me about any unexpected changes or results you saw from Breathe Easy, Live Well?**

23. **And, our last question: Is there anything else that would be useful for me to know?**

Thank you very much for taking the time to talk with me and help with the evaluation of Breathe Easy, Live Well. Your responses are very important in the development Breathe Easy, Live Well.

Conclusion

Once we finish the evaluation process in 2011, Todd will receive copies of the final evaluation report. Would you like to receive a copy, as well? [If so, request e-mail address] It will also be available on our web site [give web site]. Please feel free to call or e-mail me if you think of anything else that would be helpful for me to know.
